# Larvicidal, Repellent, and Irritant Potential of the Seed-Derived Essential oil of *Apium graveolens* Against Dengue Vector, *Aedes aegypti* L. (Diptera: Culicidae)

**DOI:** 10.3389/fpubh.2014.00147

**Published:** 2014-09-18

**Authors:** Sarita Kumar, Monika Mishra, Naim Wahab, Radhika Warikoo

**Affiliations:** ^1^Department of Zoology, Acharya Narendra Dev College, University of Delhi, New Delhi, India

**Keywords:** essential oil, larvicide, repellent, % protection, irritancy

## Abstract

*Aedes aegypti* L. is one of the primary disease vectors spreading various dreadful diseases throughout the world, specifically over tropics and subtropics. Keeping in view the adverse effects of chemical insecticides-based intervention measures, the eco-friendly and bio-degradable essential oil extracted from the seeds of celery, *Apium graveolens* were investigated for its efficacy against *Ae. aegypti*. Larvicidal bioassay carried out with the seed oil against early fourth instars of *Ae. aegypti* caused an LC_50_ and LC_90_ values of 16.10 and 29.08 ppm, respectively, after an exposure to 24 h. The cidal effect of the celery seed oil augmented by 1.2-fold; after an exposure to 48 h; revealing an LC_50_ value of 13.22 ppm. Interestingly, the seed oil did not cause immediate larval mortality, suggesting a delayed toxicity against the larval stage. Present investigations also revealed remarkable effective repellency of the oil leading to 100% protection till 165 min as compared to control that did not result in any repellency against adult *Ae. aegypti*. Interestingly, only one bite was recorded in the 165th-min after which only two bites were scored until 180 min of exposure of the adult mosquitoes to the oil. An exciting observation was that the knocked-down effect in adults exposed to 10% oil-impregnated papers. The contact irritancy assays with paper impregnated with 1% celery seed oil caused first flight only after 4 s resulting in an average of 63.66 flights during 15 min of exposure revealing the relative irritability of 26.97. The qualitative phytochemical analysis of the seed oil showed the presence of flavonoids, lactones, and terpenoids as the major constituents suggesting their probable role in the toxicity. Our results confirmed that celery seed essential oil can be used as an efficient larvicide and repellent against *Ae. aegypti*. The identification of the bioactive components, their mode of action, and studying effects on non-target organisms and the environment would help in devising mosquito-management strategies.

## Introduction

*Aedes aegypti* is considered as one of the major disease vectors that spread several dreadful diseases such as dengue fever, Chikungunya, and yellow fever worldwide specifically tropical and sub-tropical countries. In India, dengue fever is gradually becoming the most important public health problem. Every year, the number of reported dengue cases is rising. In 2013, a total of 75,454 dengue cases were reported in India, which led to 167 deaths (1). Earlier, in 2002, Pancharoen et al. (2) have reported the rising incidences of more severe forms of dengue, i.e., dengue hemorrhagic fever and dengue shock syndrome with unusual manifestations such as central nervous system involvement. The reports of World Health Organization (3) also reveal that about 40% of the world’s population is at risk of dengue. It has been suggested that the major approach to control mosquito-borne diseases should include either targeting the mosquito larvae at breeding sites or by killing/repelling the adult mosquitoes (4).

Till today, chemical insecticides have been used on a large scale to control mosquitoes at both larval and adult stage. However, the adverse effects posed by these synthetic insecticides such as non-degradability, environmental pollution, toxicity to non-target population, and the developing resistance in mosquitoes have increased during the last five decades. These adversities have insisted on the need of formulating alternate mosquito control strategies. Botanicals have always attracted researchers as an environment-friendly, safe, and low-cost alternative to chemical insecticides (5). Several reports are available, which confirm the larvicidal and repellent efficacy of plant extracts or essential oils against different species of mosquitoes without posing toxicity hazards to humans (6–10).

Essential oils, the natural volatile substances obtained from various plants, have been exploited commercially in pharmaceuticals, as flavoring agents in foods, for aroma in fragrant products, and as insecticides. The essential oils have been primarily investigated for their antibacterial, antifungal, and insecticidal activities (11–13). However, they have received great attention as probable bioactive insecticides displaying a wide-spectrum activity, low mammalian toxicity, and rapid bio-degradability.

*Apium graveolens*, commonly called celery, is an aromatic herb. The essential oil, extracted from celery fresh dried seeds through steam distillation, is used in several products of medical importance. Reports are available on the potential use of celery leaf stalks and seeds as popular aromatic herbs and spices (14, 15). Researchers have confirmed that certain bioactive components isolated from the crude alcoholic and hexane extracts of *Apium graveolens* seeds possess nematocidal activity against *Caenorhabditis elegans* and *Panagrellus redivivus*, antifungal activity against *Candida albicans*, *C. kruseii*, and *C. parapsilosis*, bactericidal activity against *Helicobacter pylori*, and mosquiticidal effects against *Ae. aegypti* fourth instars (16). However, much literature is not available on the larvicidal and repellent activities of the essential oil derived from the *Apium* seeds against *Ae. aegypti*. Hence, the present investigations were carried out to assess the larvicidal and the repellent potential of the celery seed essential oil against an Indian strain of *Ae. aegypti*. The present study may provide useful information on the bioactive component from native plant source, which could help in the development of new mosquito control agent.

## Materials and Methods

### Rearing of *Ae. aegypti*

The present investigations were carried out on the dengue fever mosquito, *Ae. aegypti*, which were collected from ponds located in Delhi, India, and surroundings. The colony of mosquito was maintained in an insectary under controlled conditions of 28 ± 1°C, 80 ± 5% RH, and 14:10 L/D photoperiod (17). Adults were kept in cloth cages and provided with freshly soaked deseeded raisins as food. A wet cotton swab was kept on the top of cage to provide water. Blood meals at regular intervals were provided to female adults for maturation of egg follicles by keeping restrained albino rats in the cages. An enamel bowl lined with Whatman filter paper was kept in the cage for egg laying. The collected eggs were allowed to hatch in trays filled with de-chlorinated tap water. Freshly hatched larvae were fed upon a 1:3 ratio mixture of yeast powder and grinded dog biscuits. The water was changed every day to prevent the formation of any scum, and larvae were provided with fresh food. The pupae formed were collected in bowls and kept in the cages for adult emergence.

### Larvicidal bioassay

The larvicidal bioassay was performed on the early fourth instars of *Ae. aegypti* following the WHO protocol with slight modifications (17). The 99.9% pure essential oil, extracted from seeds of *A. graveolens*, was obtained from M/s Auroville, Puducherry, India. Ethanol was used as the solvent to prepare the graded series of celery seed oil for bioassays. Bioassay was carried out on 20 early fourth instars of *Ae. aegypti*; taken in plastic bowls containing 99 mL of distilled water; which were then transferred to a glass jar containing 100 mL of distilled water and 1 mL of the particular concentration of oil. Each dilution had four replicates for statistical significance. Control bioassays were performed replacing the oil–ethanol solution with ethanol alone.

During the bioassays, the larvae were not provided with any food. The larval mortality was determined by observing the movement of the larvae after 24 h of treatment by touching them gently with the help of a glass rod. The larvae without any sign of movements were considered dead, while those, which moved a little but did not show any kind of swimming movement were considered moribund. The moribund larvae unable to revive were considered dead. The experimental set up was kept undisturbed for next 24 h and mortality counts were recorded again to evaluate the delayed toxicity of the essential oil.

### Statistical analysis of data

The larvicidal bioassays with more than 20% mortality in control tests and more than 20% pupae formed were discarded and carried out again. The control mortality ranging between 5 and 20% was corrected using Abbott’s formula (18).

$$\mathrm{CM =}\frac{T-C}{100-C}\times 100$$

where, CM is the corrected mortality, *T* is the % mortality observed in experimental tests, and *C* is the % mortality in control tests. The larvicidal data were subjected to probit regression analysis based on generalized linear model using computerized SPSS 18.0 Program. The regression analysis models the normal distribution of the relationship between response (% mortality) and dose (concentration) as a linear model via a link function by the transformation of % mortality in probit values. The LC_50_ and LC_90_ values with 95% fiducial limit were calculated in each bioassay to measure the difference between the test samples. Other statistical parameters, such as regression coefficient and standard error were also calculated. The fitted model is assessed by statistics for heterogeneity, which follow a chi-square distribution.

### Behavioral studies in oil-treated *Ae. aegypti* larvae

During each larvicidal bioassay, the larvae were monitored carefully for behavioral modifications, if any, caused by extract-mediated disruption of biological functions. The behavioral observations included wriggling speed, horizontal movements, vertical movements, aggregation behavior, and knockdown of the larvae during treatment. The larval behavior was recorded and photographed with Canon Power Shot SX50HS. Similar observations were made in controls for comparison with treated larvae.

### Adult repellency bioassay

The repellent potential of the celery seed oil was evaluated against adult *Ae. aegypti* using human-bait technique. Five human volunteers, non-allergic to mosquito bites, were invited from different institutes and a consent letter regarding the experiment was taken from each of them. The letters were deposited in the institute for reference, if needed. Starved and 3–10 days old females of *Ae. aegypti* were released in groups of 25 into separate laboratory cages (45 cm × 45 cm × 40 cm) for the investigations. During repellency bioassays, the arms of the human volunteers were thoroughly cleaned, washed with neutral soap without any fragrance, thoroughly rinsed with distilled water, and allowed to dry for 10 min before the application of extracts. A square of 5 cm × 5 cm size was marked on each forearm of human volunteers using a permanent marker. One forearm of all the volunteers was used for repellency bioassay and approximately 0.1 mL of the essential seed oil of *A. graveolens* was applied to the marked area. The other forearm of each volunteer was considered control and the marked area was applied with ethanol. The rest of the area of each forearm was covered by a paper sleeve thus leaving only the marked area open. Both the control and treated arms were introduced simultaneously into the cage. Any attempt of inserting the stylets by a female mosquito was considered a bite. The numbers of bites occurred in 3 min were scored every 15 min, for 3 h; from 11:00 to 14:00 hours.

Protection time was recorded as the time elapsed between the application of essential oil and the time of a confirmed bite. The tests, with none of the adult *Ae. aegypti* landing on the control arm or attempting to bite, were discarded. These tests were carried out again with a fresh batch of adults to ensure that failure to bite was due to repellence potential of oil and not because of the mosquitoes being pre-disposed to get a blood meal. Three replicates of each experiment were carried out. Each replicate was conducted in separate cages and with different volunteers to negate the effect of skin variability on repellency, if any.

The percent protection from the mosquito bite provided by the oil was calculated by using the following formula:

$$\%\text{ Protection}\;=\;\frac{\begin{array}{c} \text{Number of bites on the control arm}-\\ \;\text{Number of bites on the treated arm} \end{array}}{\text{Number of bites on the control arm}}\times 100$$

### Contact irritancy assay

The contact irritancy assay was performed on 3-day-old non-blood females of *Ae. aegypti*. The oil-impregnated papers were prepared with Whatman filter paper no. 1, which were cut out in circles of 8 cm diameter. The papers were impregnated with 10 and 1% of celery seed essential oil and then allowed to shade-dry. The completely dried paper was placed on a glass plate, and a perspex funnel with a hole on the top was kept inverted over the impregnated paper. Single female adult was released in the funnel and per-conditioned for 3 min. Thereafter, the time at which first flight was taken was recorded. The experiment was continued for 15 min and the total number of flights undertaken by each female adult was scored. Three replicates were carried out for each treatment. Parallel control tests were performed with papers impregnated with acetone. The relative irritability caused by the seed oil was calculated with respect to control by the following formula:

$$\text{RI }\left(\text{Relative Irritability}\right)=\frac{\begin{array}{c} \text{Mean number of take-offs}\\ \;\text{stimulated by the oil} \end{array}}{\begin{array}{c} \text{Mean number of take-offs}\\ \;\text{stimulated by control} \end{array}}$$

### Phytochemical analysis

The essential oil extracted from *A. graveolens* seeds was analyzed for the presence of phytochemical components using standard procedures as illustrated by Harborne (19). The qualitative biochemical assays were performed to identify the presence of secondary metabolites; alkaloids, carbohydrates, flavonoids, phenolic compounds, phlobatannins, saponins, tannins, and terpenoids.

## Results

The potential of essential oil extracted from the seeds of celery plant, *A. graveolens* was evaluated as larvicidal and repellent agent against dengue vector, *Ae. aegypti*. The investigations validated the significant potential of essential oil as the probable agent for the control and management of rising *Ae. aegypti* population.

### Larvicidal bioassay

The 24 h exposure of early fourth instars of *Ae. aegypti* to the essential seed oil of *A. graveolens* resulted in quite low LC_50_ and LC_90_ values of 16.10 and 29.08 ppm, respectively (Table 1). The toxicity potential of the oil increased by 1.2-fold (LC_50_ = 13.22 ppm) on exposure of the larvae to the oil for another 24 h. The bioassay did not cause the formation of pupa or larval–pupal intermediates resulting in complete mortality of the larvae. The control treatments did not cause any mortality till 48 h (Table 1).

**Table 1:** **Larvicidal activity of the essential oil derived from *Apium graveolens* seeds against early fourth instars of *Aedes aegypti***.

	Larvicidal activity		Regression coefficient ± SE	χ^2^ (df)	*p* Value
	LC_50_	LC_90_			
Exposure to 24 h	16.10 (11.93–22.08)	29.08 (21.40–64.61)	4.99 ± 1.36	0.79 (3)	0.8519
Exposure to 48 h	13.22 (9.80–18.70)	28.14(19.64–60.78)	3.90 ± 0.87	1.27 (3)	0.7363

### Behavioral studies in oil-treated *Ae. aegypti* larvae

The larvae were scrutinized carefully during the treatment period for any behavioral modifications. The observations revealed that *A. graveolens* oil did not cause immediate or quick mortality. Initial exposure of the larvae to the essential oil did not affect the larvae and all larvae were found moving normally and exhibited a typical appearance. The restlessness in the larval behavior was noticed after 10 min of treatment. The principal lethal effects of the seed oil were observed after approximately 20–25 min of treatment in the form of incapability of rising to the water surface, body tremors, and convulsions. The symptoms of paralysis were clearly visible in 20% of the larvae after an hour leading to death of these larvae. Continued exposure of the larvae to oil caused mortality in approximately 50% larvae after 5–6 h, and the death of most of the larvae was observed within 10 h.

### Adult repellency bioassay

Investigations conducted on the repellency potential of celery seed oil against adults *Ae. aegypti* revealed it as a promising and notable repellent. The oil resulted in 100% protection against bites by female *Ae. aegypti* in the first 150 min as compared to the ethanol that did not cause any repellency against mosquito bites (Table 2). When the experiment was continued for next 15 min, only one bite was recorded as compared to the nine bites scored on the control arm. It clearly reveals the reduced protection from 100 to 88.8% in the 165th-min. The percent protection to adult *Aedes* further decreased to 77% with two bites recorded after 180 min of exposure to the oil (Table 2). It is significant to note that direct application of the *A. graveolens* oil did not induce any dermal irritation during the experiment as well as afterward.

**Table 2:** **Percent repellency and percent protection to the bites of *Aedes aegypti* after application of the essential oil of *Apium graveolens* on the arms of human volunteers**.

Time (min)	Mean no. of mosquito bites		% Repellency after oil application	% Protection after oil application
	Control	Celery seed oil	
15	3.0	0.0	100.0	100.0
30	5.67	0.0	100.0	100.0
45	3.33	0.0	100.0	100.0
60	3.0	0.0	100.0	100.0
75	7.67	0.0	100.0	100.0
90	8.33	0.0	100.0	100.0
105	7.33	0.0	100.0	100.0
120	5.67	0.0	96.0	82.4
135	6.33	0.0	100.0	100.0
150	7.67	0.0	100.0	100.0
165	9.00	1.0	96.0	88.8
180	8.67	2.0	92.0	76.9

### Contact irritancy assay

A significant elicit response was observed in the adults of *Ae. aegypti* when subjected to contact irritancy assays. The exposure to 10% oil led to complete knockdown of adults when released for acclimatization in the funnel for 3 min. Conversely, exposure to 1% seed oil caused first flight of the female adult after a mean time of 4 s (Table 3). The average total of 63.66 take-offs were observed after 15 min of exposure to 1% oil as compared to only 2.36 flights when exposed to ethanol-impregnated paper resulting in the relative irritability of 26.97.

**Table 3:** **Response of 3-day-old adult females of *Aedes aegypti* in the contact irritancy assays to celery seed essential oil-impregnated papers**.

Impregnated paper	Mean time lapse before first take-off (min)	Mean number of take-offs for females (in 15 min)	Relative irritability
Control	7.05 ± 0.72^a^	2.36 ± 0.72	1.0
10% oil	Knockdown	–	–
1% oil	0.04 ± 1.66	63.66 ± 0.66	26.97

*^a^Mean ± SEM*.

### Phytochemical analysis

The qualitative biochemical analysis for the secondary metabolites present in the essential oil of *A. graveolens* seeds revealed the presence of terpenoids, lactones, and flavonoids as the main constituents. Other tested components were not detected in the essential seed oil of *A. graveolens* (Table 4).

**Table 4:** **Phytochemical screening of the essential oil of the seeds of *Apium graveolens***.

S. No.	Plant constituents	Celery seed essential oil
1	Alkaloid	−
2	Carbohydrates	−
3	Saponins	−
4	Phenolic compounds	−
5	Tannins	−
6	Flavonoids	+
7	Terpenoids	+
8	Phlobatannins	−
9	Lactones	+

## Discussion

Mosquito-borne diseases are increasing each year in tropical and sub-tropical countries. Since many decades, chemical insecticides have been used to combat the mosquito menace. However, the continued and frequent use of these insecticides has caused adverse effects, including toxicity to non-target organisms and humans, environment pollution, and increased development of resistance in the mosquito population. Botanicals have now become favorite agents among researchers as suitable alternatives to the toxic chemical insecticides. A few reports are available regarding the larvicidal and repellent potency of essential oils, volatiles extracted from different plants, against *Ae. aegypti* (20–22).

Keeping these in view, present studies were conducted to assess the probable role of celery seed oil as larvicidal, repellent, and irritancy agent for the control and management of *Ae. aegypti* population. Our investigations on early fourth instars of *Ae. aegypti* showed that 24 h of exposure to the oil resulted in an LC_50_ and LC_90_ value of 16.10 and 29.08 ppm, respectively (*p* > 0.05). It was also revealed that when the larvae were exposed to the oil for 48 h, the toxicity potential of the oil rose by 1.2-fold. However, keeping in view the large population of *Ae. aegypti* and enormous heterogeneity in their population; the chi-square distribution and insignificant *p* values obtained in our investigations suggest bioassays with more random selection of larvae and increased number of replicates to confirm the larvicidal potential of celery seed oil. Similar larvicidal activity of the ethanol-extracted *A. graveolens* was reported by Choochote et al. (23) against a Thailand strain of *Ae. aegypti*, the fourth instars exhibiting LD_50_ and LD_95_ values of 81.0 and 176.8 mg/L, respectively. The significant larvicidal activity of the volatile oils of *A. graveolens* has also been reported by Pitasawat et al. (24) against the two mosquito species, *Ae. aegypti* and *Anopheles stephensi* after an exposure to 24 h.

Several researchers have evaluated the larvicidal potential of various other essential oils against mosquitoes. The excellent larvicidal efficiency of the essential oil extracted from *Mentha piperita* revealing an LC_50_ and LC_90_ value of 111.9 and 295.18 ppm, respectively after 24 h of exposure has been reported against dengue vector (8). They also showed that the toxicity of the peppermint oil increased by 11.8% on exposure to the oil for 48 h. Similarly, Warikoo et al. (25) observed essential oil isolated from *Pinus longifolia* as the efficient larvicidal agent against *Ae. aegypti*. On exposure to commercially available pine oil, the early fourth instar larvae showed an LC_50_ value of 0.330 mg L^−1^ and an LC_90_ value of 1.118 mg L^−1^. Recently, Liu et al. (10) established the larvicidal potential of essential oil derived from the roots of *Toddalia asiatica* and the constituents isolated from the oil against *Ae. albopictus*. The essential oil extracted from the leaves of *Feronia limonia* showed remarkable larvicidal activity against *An. stephensi* with LC_50_ value of 15.03 ppm after 24 h, while against *Ae. aegypti* and *Cx*. *quinquefasciatus*, the LC_50_ values reported were 11.59 and 22.49 ppm, respectively (26). Lee (27) evaluated the larvicidal activity of essential oils derived from 11 aromatic medicinal plants against early fourth instars of *Ae. aegypti* and reported 100% mortality on exposure to all oils at 100 ppm. Cheng et al. (28) investigated the larvicidal potential of the essential oils from the leaves of eight provenances of indigenous cinnamon (*Cinnamomum osmophloeum* Kaneh.) and reported the excellent inhibitory effect of the essential oils of cinnamaldehyde type and cinnamaldehyde/cinnamyl acetate type against the IV instars of *Ae. aegypti*. Choochote et al. (23) have observed significant larvicidal activity of the volatile oils of *Curcuma aromatica* against the fourth instars of *Ae. aegypti* with an LC_50_ value of 36.30 ppm. Pushpanathan et al. (29) reported the LC_50_ values of 50.78 ppm when the essential oil extracted from *Zingiber officinalis* was tested against *Cx. quinquefasciatus*. Tiwary et al. (30) tested the essential oil of *Zanthoxylum armatum* against three species of mosquitoes and reported *Cx. quinquefasciatus* to be the most susceptible against oil with an LC_50_ value of 49 ppm followed by *Ae. aegypti* and *An. stephensi* with LC_50_ values in the range of 54–58 ppm.

Present study demonstrated the delayed toxicity of *A. graveolens* oil against early fourth instars of *Ae. aegypti* instead of quick larval mortality. Similarly, Choochote et al. (23) suggested the delayed larval killing effect of the ethanol-extracted celery seed oil *A. graveolens* against *Ae. aegypti*. The larvae treated with celery seed essential oil exhibited excitation and aggressive vertical and horizontal movements. These symptoms suggest the probable impact of oil on the neuro-muscular co-ordination in chemical synapses. These findings are in conformity with few earlier studies (17, 23, 31).

The exposure of the adults *Ae. aegypti* to celery seed oil established the promising and remarkable repellency potential. The oil provided 100% protection to human volunteers in the first 150 min followed by only one to two bites in the next 30 min of exposure. Likewise, the crude seed extract of celery has been reported to exhibit repellent activity against *Ae. aegypti* with ED_50_ and ED_95_ values of 2.03 and 28.12 mg/cm^2^, respectively, providing the biting protection time of 3 h on application at a concentration of 25 g% (23). The mosquito repellent potential of celery, *A. graveolens*, has also been compared with commercial repellents by Tuetun et al. (32). Kumar et al. (8) reported the repellent properties of essential oil extracted from *M. piperita* against adults *Ae. aegypti* with 100% protection till 150 min after which only one to two bites were recorded during the next 30 min, as compared to eight to nine bites on the control arm. The skin repellent tests performed at 1.0, 2.0, 3.0, and 4.0 mg/cm^2^ with essential oil extracted from *Z. officinalis* gave 100% protection against *Cx. quinquefasciatus* up to 120 min (29). The repellent activities of the essential oil of *Cinnamomum zeylanicum*, *Z. officinale*, and *Rosmarinus officinalis* have been also reported by Prajapati et al. (33) against *An. stephensi, Ae. aegypti*, and *Cx. quinquefasciatus*.

In the present investigations, the contact irritancy assays with the celery seed oil resulted in a significant elicit response in the adults of *Ae. aegypti*. Exposure to 1% oil led to average number of 63.66 take-offs in 15 min with first flight after a mean time of 4 s. The behavioral modifications in the mosquitoes through chemical actions in contact irritancy may diminish population that may ultimately reduce disease transmission (34). Exposure of the female adults of *Ae. aegypti* to the crude leaf extracts of *Parthenium hysterophorus* showed a similar significant repellency behavior (9). Nour et al. (35) suggested the utilization of 0.1% essential oils of *Ocimum basilicum* as promising natural repellents against *Anopheles* mosquito.

Studies have shown that secondary metabolites; steroids, alkaloids, terpenoids, saponins, phenolics, essential oil, etc., of plants play a major role in the mosquito control being associated with a wide range of bioefficacy. The phytochemical analysis of the essential oil of *A. graveolens* seeds showed the presence of terpenoids, lactones, and flavonoids as the main constituents. Fazal and Singla (36) have reported d-limonene (80%) as the prime constituent in celery seed oils. They have also reported selinene, various sesquiterpene alcohols, b-elemne, linalool, *N*-butylphthalide, sedanenolide, and sedanonic anhydride as the other secondary metabolites in celery seed oil. However, the larvicidal and repellency potential of the essential celery seed oil reported in the present study need to be further investigated for identification of the compound responsible. It is further suggested that the efficacy observed may be because of the individual or the synergistic effects of various compounds present in them, identified or unidentified.

The potential role of *A. graveolens* seed essential oil as larvicide and repellent has been investigated against *Ae. aegypti*. Nevertheless, this is an explorative evaluation and the studies have suggested the potential bioefficacy of celery seed oil. However, keeping in view the heterogeneous and large population of *Ae. aegypti*, further complex investigations with more random selection of larvae and increased number of replicates are needed to ascertain the efficacy of celery seed oil against *Ae. aegypti*. Moreover, the identification of bioactive components present in the oil and understanding their mode of action is essential for its use as mosquito control agent. Field trials are recommended before the use of *A. graveolens* seed oil as an anti-mosquito natural, environment-friendly product in the mosquito-management program.

## Conflict of Interest Statement

The authors declare that the research was conducted in the absence of any commercial or financial relationships that could be construed as a potential conflict of interest.

## References

[1] National Vector Borne Disease Control Programme. Dengue Cases and Deaths in the Country since 2007. (2014). Available from: http://nvbdcp.gov.in/den-cd.html

[2] Pancharoen C Kulwichit W Tantawichien T Thisyakorn U Thisyakorn C Dengue infection: a global concern. J Med Assoc Thailand (2002) 85:S23–33 12188420

[3] World Health Organization. Dengue and Dengue Haemorrhagic Fever. (2009). Available from: http://www.who.int/mediacentre/factsheets/fs117/en/

[4] Joseph CC Ndoile MM Malima RC Nkuniya MHM Larvicidal and mosquitocidal extracts, a coumarin, isoflavonoids and pterocarpans from *Neorautanenia mitis*. Trans Royal Soc Trop Med Hyg (2004) 98:451–5 10.1016/j.trstmh.2003.10.008 15186932

[5] Sukumar K Perich JM Boobar RL Botanical derivatives in mosquito control: a review. J Am Mosq Contr Assoc (1991) 7:210–37 1680152

[6] Rahuman AA Bagavan A Kamaraj C Vadivelu M Zahir AA Elango G Evaluation of indigenous plant extracts against larvae of *Culex quinquefasciatus* Say (Diptera: Culicidae). Parasitol Res (2009) 104:637–43 10.1007/s00436-008-1240-9 18975001

[7] Rahuman AA Bagavan A Kamaraj C Saravanan E Zahir AA Elango G Efficacy of larvicidal botanical extracts against *Culex quinquefasciatus* Say (Diptera: Culicidae). Parasitol Res (2009) 104:1365–72 10.1007/s00436-009-1337-9 19198882

[8] Kumar S Wahab N Warikoo R Bioefficacy of *Mentha piperita* essential oil against dengue fever mosquito, *Aedes aegypti* L. Asia Pacific J Trop Biomed (2011) 2:90–3 10.1016/S2221-1691(11)60001-4 23569733 PMC3609176

[9] Kumar S Singh AP Nair G Sahil B Wahab N Warikoo R Impact of *Parthenium hysterophorus* leaf extracts on the fecundity, fertility and behavioural response of *Aedes aegypti* L. Parasitol Res (2011) 108:853–9 10.1007/s00436-010-2126-1 20978787

[10] Liu XC Dong HW Zhou L Du SS Liu ZL Essential oil composition and larvicidal activity of *Toddalia asiatica* roots against the mosquito *Aedes albopictus* (Diptera: Culicidae). Parasitol Res (2013) 112:1197–203 10.1007/s00436-012-3251-9 23271568

[11] Franzios G Morotson M Hatziapostolou E Kral J Scouras ZG Mavragani TP Insecticidal and genotoxic activities of mint essential oils. J Agric Food Chem (1997) 45:2690–4 10.1021/jf960685f

[12] Chang ST Wang SY Wu CL Chen PF Kuo YH Comparisons of the antifungal activities of cadinane skeletal sesquiterpenoids from Taiwania (*Taiwania cryptomerioides* Hayata) heart-wood. Holzforschung (2000) 54:241–5 10.1515/HF.2000.041

[13] Chang ST Chen PF Chang SC Antibacterial activity of essential oils and extracts from Taiwania (*Taiwania cryptomerioides* Hayata). Q J Chin Forest (2000) 33:119–25

[14] Rafikali AM Muraleednaran GN Mosquitocidal, nematicidal and antifungal compounds from *Apium graveolens* L. seeds. J Agric Food Chem (2001) 49:142–5 10.1021/jf001052a 11305251

[15] Kitajima J Ishikawa T Satoh M Polar constituents of celery seed. Phytochemistry (2003) 64:1003–11 10.1016/S0031-9422(03)00461-8 14561518

[16] Saini N Singh GK Nagori BP Spasmoltic potential os some medicinal plants belonging to Family Umbelliferae: a review. Int J Res Ayurveda Pharm (2014) 5:74–83 10.7897/2277-4343.05116

[17] Kumar S Warikoo R Wahab N Larvicidal potential of ethanolic extracts of dried fruits of three species of peppercorns against different instars of an Indian strain of dengue fever mosquito, *Aedes aegypti* L. (Diptera: Culicidae). Parasitol Res (2010) 107:901–7 10.1007/s00436-010-1948-1 20549234

[18] Abbott WB A method for computing the effectiveness of an insecticide. J Econ Entomol (1925) 18:265–7

[19] Harborne JB Phytochemical Methods: A Guide to Modern Techniques of Plant Analysis. London, UK: Chapman & Hall (1998). 302 p.

[20] Morais SM Eveline SB Cavalcanti ESP Luciana MB Oliveira CLL Rodrigues JRB Larvicidal activity of essential oils from Brazilian *Croton* species against *Aedes aegypti* L. J Am Mosq Contr Assoc (2006) 22:161–4 10.2987/8756-971X(2006)22[161:LAOEOF]2.0.CO;2 16646345

[21] Silva WJ Doria GA Maia RT Nunes RS Carvalho GA Blank AF Effects of essential oils on *Aedes aegypti* larvae: alternatives to environmentally safe insecticides. Biores Technol (2008) 99:3251–5 10.1016/j.biortech.2007.05.064 17662602

[22] Waliwitya R Kennedy CJ Lowenberger CA Larvicidal and oviposition-altering activity of monoterpenoids, trans-anithole and rosemary oil to the yellow fever mosquito *Aedes aegypti* (Diptera: Culicidae). Pest Manag Sci (2009) 65:241–8 10.1002/ps.1675 19086001

[23] Choochote W Tuetun B Kanjanapothi D Rattanachanpichai E Chaithong U Chaiwong P Potential of crude seed extract of celery, *Apium graveolens* L. against the mosquito *Aedes aegypti* (Diptera: Culicidae). J Vector Ecol (2004) 29:340–6 15707293

[24] Pitasawat B Champakaew D Choocote W Jitpakdi A Chaithong U Kanjanapothi D Aromatic plant-derived essential oil: an alternative larvicide for mosquito control. Fitoterapia (2007) 78:205–10 10.1016/j.fitote.2007.01.003 17337133

[25] Warikoo R Wahab N Kumar S Larvicidal potential of commercially available pine (*Pinus longifolia)* and cinnamon (*Cinnamomum zeylanicum*) oils against an Indian strain of dengue fever mosquito, *Aedes aegypti* L. (Diptera: Culicidae). Acta Entomol Sin (2011) 54:793–9

[26] Senthilkumar A Jayaraman M Venkatesalu V Chemical constituents and larvicidal potential of *Feronia limonia* leaf essential oil against *Anopheles stephensi*, *Aedes aegypti* and *Culex quinquefasciatus*. Parasitol Res (2012) 112:1337–42 10.1007/s00436-012-3188-z 23160893

[27] Lee H-S Mosquito larvicidal activity of aromatic medicinal plant oils against *Aedes aegypti* and *Culex pipiens pallens*. J Am Mosq Contr Assoc (2006) 22:292–5 10.2987/8756-971X(2006)22[292:MLAOAM]2.0.CO;2 17019775

[28] Cheng S-S Liu J-Y Tsai K-H Chen W-J Chang S-T Chemical composition and mosquito larvicidal activity of essential oils from leaves of different *Cinnamomum osmophloeum* Provenances. J Agric Food Chem (2004) 52:4395–400 10.1021/jf0497152 15237942

[29] Pushpanathan T Jebanesen A Govindarajan M The essential oil of *Zingiber officinalis* Linn (Zingiberaceae) as a mosquito larvicidal and repellent agent against the filarial vector *Culex quinquefasciatus* Say (Diptera: Culicidae). Parasitol Res (2008) 102:1289–91 10.1007/s00436-008-0907-6 18278511

[30] Tiwary M Naik SN Tewary DK Mittal PK Yadav S Chemical composition and larvicidal activities of the essential oil of *Zanthoxylum armatum* DC (Rutaceae) against three mosquito vectors. J Vector Borne Dis (2007) 44:198–204 17896622

[31] Chaithong U Choochote W Kamsuk K Jitpakdi A Tippawangkosol P Chaiyasit D Larvicidal effect of pepper plants on *Aedes aegypti* (L.) (Diptera: Culicidae). J Vector Ecol (2006) 31:138–43 10.3376/1081-1710(2006)31[138:LEOPPO]2.0.CO;2 16859102

[32] Tuetun B Choochote W Kanjanapothi D Rattanachanpichai E Chaithong U Chaiwong P Repellent properties of celery, *Apium graveolens*, compared with commercial repellents, against mosquitoes under laboratory and field conditions. Trop Med Int Health (2005) 10:1190–8 10.1111/j.1365-3156.2005.01500.x 16262746

[33] Prajapati V Tripathi AK Aggarwal KK Khanuja SPS Insecticidal, repellent and oviposition-deterrent activity of selected essential oils against *Anopheles stephensi*, *Aedes aegypti* and *Culex quinquefasciatus*. Biores Technol (2005) 96:1749–57 10.1016/j.biortech.2005.01.007 16051081

[34] Said SH Grieco JP Achee NL Evaluation of contact irritant and spatial repellent behavioural responses of male *Aedes aegypti* to vector control compounds. J Am Mosq Contr Assoc (2009) 25:436–41 10.2987/09-5895.1 20099590

[35] Nour AH Elhussein SA Osman NA Nour AH Repellent activities of the essential oils of four Sudanese accessions of basil (*Ocimum basilicum* L.) against *Anopheles* mosquito. J Appl Sci (2009) 9:2645–8 10.3923/jas.2009.2645.2648

[36] Fazal SS Singla RK Review on the pharmacognostical and pharmacological characterization of *Apium graveolens* linn. Indo Global J Pharma Sci (2012) 2:36–42

